# Enhancing Monitoring of Clozapine-Related Constipation in Sunstone Ward, Longer-Term High Dependency Rehabilitation Unit

**DOI:** 10.1192/bjo.2026.11256

**Published:** 2026-06-30

**Authors:** Ikrom Arzikulov, Daniel Hughes

**Affiliations:** North London NHS Foundation Trust, London, United Kingdom

## Abstract

**Aims:**

Clozapine is the most effective treatment for treatment-resistant schizophrenia but is associated with a high risk of constipation which can lead to serious and potentially fatal complications if not recognised and managed early. This Quality Improvement Project (QIP) aimed to enhance the monitoring of clozapine-related constipation on Sunstone Ward by increasing the proportion of clozapine-treated patients with a fully completed Bristol Stool Chart (BSC) and constipation symptom screening documentation to 100% by mid December 2025.

**Methods:**

This QIP was conducted on Sunstone Ward, a 17-bed male high-dependency rehabilitation unit. Baseline data were collected in April 2025 through retrospective review of clinical documentation for clozapine-treated patients.

Four Plan–Do–Study–Act (PDSA) cycles were implemented between June and December 2025. Interventions included daily assigned nurse-led bowel movement assessments using the BSC, structured documentation prompts, routine review of BSCs during ward rounds, increased patient engagement in reporting bowel habits, and a targeted staff teaching session on clozapine-related constipation.

Outcome measures were the percentage of clozapine-treated patients with fully completed BSCs and constipation symptom screening documentation.

**Results:**

Baseline data showed poor monitoring, with only 25% of patients having a fully completed BSC and 37.5% having documented symptom screening. Following PDSA Cycle 1, modest improvements were observed (BSC 50%, symptom screening documentation 62.5%). In PDSA Cycle 2, BSCs were routinely reviewed during ward rounds, leading to marked improvement in compliance (both measures 87.5%). PDSA Cycle 3 focused on patient engagement, resulting in BSC completion reaching 100%, although symptom screening documentation plateaued at 87.5%. Following a targeted staff teaching session in PDSA Cycle 4, both BSC completion and symptom screening reached and were sustained at 100% by mid December 2025.

**Conclusion:**

This QIP demonstrated that clozapine-related constipation monitoring can be significantly improved through daily assigned nurse-led bowel assessments, structured documentation prompts, routine review of BSCs during ward rounds, patient engagement and staff education. While early interventions improved compliance, patient engagement and staff teaching were key to achieving full and sustained monitoring. Embedding bowel monitoring into routine inpatient care enhances patient safety and reduces the risk of seriouscomplications. The approach is sustainable and transferable to other inpatient mental health settings whereclozapineis prescribed.

